# Fibroblast–Neuron interactions Driving persistent Pain in Rheumatoid Arthritis (FiND-Pain RA) – an observational study protocol

**DOI:** 10.1136/bmjopen-2024-097892

**Published:** 2025-12-25

**Authors:** Mikalena Xenophontos, Friederike C Baldeweg, Rosie Ross, Zoe Rutter-Locher, Sarah Hill, Sarah Ryan, Mosab Ali Awadelkareem, Shing T Law, David L Bennett, Christopher D Buckley, Frances Humby, Bruce W Kirkham, Franziska Denk, Leonie S Taams

**Affiliations:** 1 Kennedy Institute of Rheumatology, University of Oxford, Oxford, UK; 2 Nuffield Department of Orthopaedics Rheumatology and Musculoskeletal Sciences, University of Oxford, Oxford, UK; 3 Centre for Inflammation Biology and Cancer Immunology (CIBCI), King’s College London, London, UK; 4 Department of Rheumatology, Guy's and St Thomas’ NHS Foundation Trust, London, UK; 5 Nuffield Department of Clinical Neurosciences, University of Oxford, Oxford, UK; 6 Wolfson Sensory, Pain and Regeneration Centre (SPaRC), King’s College London, London, UK

**Keywords:** RHEUMATOLOGY, IMMUNOLOGY, Chronic Pain, Biopsy

## Abstract

**Abstract:**

**Introduction:**

Pain in patients with rheumatoid arthritis (RA) is an unmet clinical need. Targeting joint inflammation with disease-modifying antirheumatic drugs has not resulted in the anticipated reduction in pain for many patients. This can partly be explained by the concept of central sensitisation whereby spinal and supraspinal pathways have a lower threshold of activation, leading to increased perception of pain. Synovial stromal cells, such as fibroblasts, are also thought to play a role through peripheral sensitisation of nerves in the joint. Synovial fibroblasts are known to produce pro-algesic mediators such as interleukin 6 and nerve growth factor at the messenger RNA level. These pro-algesic mediators could activate sensory nerve fibres that send signals from the joint to the spinal cord, thereby driving persistent pain in RA. The purpose of this study is to evaluate which pro-algesic mediators are produced by lining versus sub-lining fibroblasts and whether the level of these mediators correlates with clinical measures of pain in patients with RA.

**Methods and analysis:**

FiND-Pain RA is a multicentre observational study which will recruit 50 patients with seropositive RA who attend the rheumatology department of Guy’s and St Thomas’ Hospital, London, and the Nuffield Orthopaedic Centre, Oxford. Clinical examination, pain-focused patient-reported outcome measures, ultrasound examination and ultrasound-guided synovial biopsy of the knee will be performed. The levels of known and putative pro-algesic mediators will be measured in fibroblasts from the lining and sub-lining layer of the synovium. The location and spatial morphology of sensory nerve fibres and their proximity to lining and sub-lining fibroblasts will be characterised. The primary outcome will be to determine whether the knee pain scores of participants correlate with the level of leukaemia inhibitory factor, a novel putative pain-mediator expressed in sub-lining fibroblasts. The secondary outcomes will be to determine whether other pro-algesic mediators produced by lining or sub-lining fibroblasts correlate with clinical measures of pain and to assess the location and proximity of sensory nerve fibres to lining versus sub-lining fibroblasts.

**Ethics and dissemination:**

The study is a sub-study of the PUMIA (Pain Phenotypes and their Underlying Mechanisms in Inflammatory Arthritis) study, which has been approved by the Bromley Research Ethics Committee (REC: 21/LO/0712). The findings of this study will be disseminated through open-access publications, as well as scientific and clinical conferences.

**STRENGTHS AND LIMITATIONS OF THIS STUDY**

- Participants will undergo comprehensive pain phenotyping using pain-focused patient-reported outcome measures and pressure algometry, improving our understanding of the peripheral drivers of pain.
- Collection of matched blood, synovial fluid and/or synovial tissue biopsies will aid future biomarker discovery efforts.
- Methods and study design were cross-checked by our collaborative team, ensuring an interdisciplinary neuro-immune-stromal perspective on pain in rheumatoid arthritis.
- Ultrasound-guided synovial tissue biopsies are small, which is particularly challenging for nerve fibre imaging.
- Our stringent inclusion/exclusion criteria will favour a homogeneous cohort but might also pose recruitment challenges.

## Introduction

### Background

 Rheumatoid arthritis (RA) is an immune-mediated inflammatory disease which causes pain, joint damage and subsequent disability.^1 2^ Pain has been ranked as the highest priority for improvement by patients with RA, yet remains an unmet clinical need.^3^ The use of biological and targeted therapies has reduced joint inflammation and damage; however, it has not improved pain to the same extent.^4 5^ Overall, 75% of European and 82% of US patients reported moderate to severe persistent pain even though they felt that their RA was ‘somewhat to completely’ controlled.^6^

Traditionally, pain has been attributed to joint inflammation in patients with RA. Sensory nerve fibres are known to be present in the deeper layers of the synovium in RA, now commonly referred to as sub-lining.^7 8^ Pro-inflammatory cytokines in the joint, such as interleukin 6 (IL-6) and tumour necrosis factor (TNF)-alpha, have their corresponding receptors on these sensory nerve fibres and are thought to initiate noxious signalling which is relayed via nociceptive pathways in the spinal cord to the brain where they can be perceived as pain.^9^ However, given that pain frequently does not resolve fully with biological therapies targeting such cytokines, there must be alternative drivers of pain which are uncoupled from ‘classical’ synovial tissue inflammation.^10 11^

Central sensitisation is widely accepted by rheumatologists as a mechanism whereby pain is potentiated and amplified through the phenomenon of neurons within central nervous system nociceptive pathways responding more readily to ordinary levels of sensory neuron input.^12^ Fibromyalgia is a condition thought to be caused by central sensitisation and is common among patients with moderate to severe RA, both in the presence and absence of inflammation.^13^ However, fibromyalgia is not the sole explanation for persistent pain in the absence of traditional measures of joint inflammation. The Testing and Identifying Targets in Rheumatoid Arthritis ThErapy-UltraSound (TITRATE-US) study recently described that 27% of patients with moderate to severe RA did not meet the American College of Rheumatology (ACR) 2010 criteria for fibromyalgia and displayed no evidence of joint inflammation, as measured by ultrasound examination of the joints.^13^ This points to alternate mechanisms driving persistent pain in RA.

One potential option is the presence of inflammation caused by dysregulation of tissue resident cells, such as fibroblasts, which are well-known for driving and maintaining joint pathology in RA.^14^,^16^ Recent work suggests that fibroblasts may also be involved in the generation of pain. For example, synovial fibroblasts activated by TNF-alpha can promote neuronal sensitisation in an in vitro model.^17^ Furthermore, in a clinical setting, it has been reported that a gene expression module, consisting of 815 genes over-represented in lining layer fibroblasts, correlates with pain in the presence of low inflammation in RA synovial biopsy samples.^18^ Finally, our group has re-examined publicly available single cell transcriptomic data from synovial biopsies from individuals with RA.^19^,^21^ This analysis demonstrated that fibroblasts in the sub-lining layer of the synovium are particularly enriched for both known and putative pro-algesic mediators such as nerve growth factor (NGF),^22^ IL-6,^23^ complement C3,^24^ leukaemia inhibitory factor (LIF)^25^ and IL-11.^26^ C3, LIF and IL-11 are considered putative pro-algesic mediators based on messenger RNA expression of their receptors (C3AR1, CR2, LIFR and IL11RA) on sensory neurons and prior literature.^24 25^ Moreover, we have recently shown that both LIF and IL-11 can induce intracellular activation of human stem-cell derived sensory neurons.^26^

**Figure 1:**
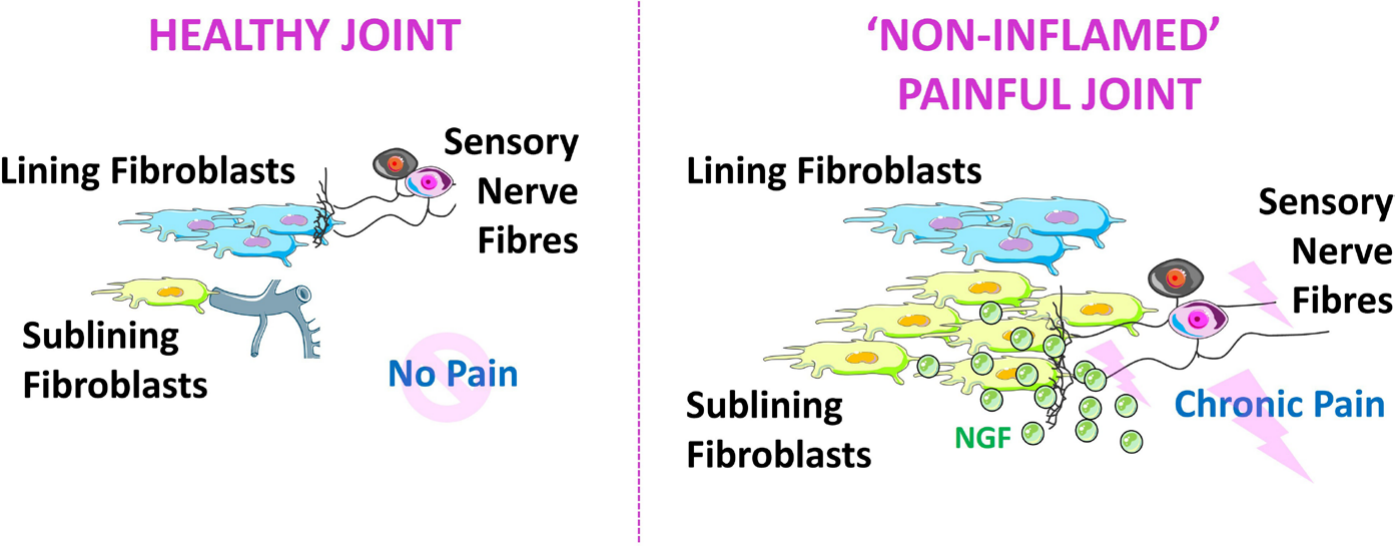
Schematic representation of how perivascular sub-lining fibroblasts may be driving pain in rheumatoid arthritis. Pro-algesic mediators such as nerve growth factor (NGF) produced by sub-lining fibroblasts attract sensory nerve fibres to the sub-lining layer. Sensory nerve fibres become activated and signal via the nociceptive pathways to the spinal cord and brain where pain is perceived. This figure was, in part, compiled using images from Servier Medical Art under a CC BY 4.0 licence.

Intriguingly, in RA, there appears to be an expansion of perivascular sub-lining THY1+ fibroblasts and a concomitant retraction of sensory nerve fibres from the lining into the sub-lining layer.^7 27^ It is thus feasible that nerve fibres are attracted to move back into the sub-lining by perivascular and fibroblast-derived neurotrophic mediators such as NGF (figure 1). Altogether, the currently available evidence leads us to hypothesise that fibroblasts, and possibly other stromal cells, in the synovium of patients with RA are key drivers of persistent pain.

### Objectives

FiND-Pain RA aims to understand whether synovial fibroblasts produce pro-algesic mediators which could be driving pain in RA.

## Methods and study design

### Patient and public involvement

Individuals with lived experience have informed this study from its very inception. This interaction started in 2018, when LST and FD hosted a workshop designed to generate new research ideas in the neuro-immune space. During discussions with academics, including BWK, several patient partners argued that more studies should be conducted on pain, since this is such a persistent problem in RA. We have worked with them and other members of the King’s College London Musculoskeletal Expert Patient Group extensively since, co-producing training workshops for students as well as publishing papers.^28^,^30^ Patient partners were also involved in preparing the Wellcome Trust Collaborative Award application that is funding the current study. In terms of engagement, we recently held an event at King’s College London where we discussed the work described in this protocol and showed a group of patients the lab including the cell sorter and cultured fibroblasts. We will continue to disseminate our findings through similar events, as well as liaise with the expert patient group at King’s College London and the Oxford Patient Engagement Network for Arthritis and Musculoskeletal Conditions.

### Study design

This is a multicentre observational study which will recruit 50 participants with established seropositive RA who meet the ACR / European League Against Rheumatism(EULAR) 2010 criteria; we will assess their disease activity and phenotype their pain. Clinical examination, pain-focused patient-reported outcome measures (PROMs), ultrasound examination and ultrasound-guided synovial biopsy of the knee will be carried out. In the laboratory, synovial tissue biopsies will be digested, and sub-lining and lining fibroblasts will be sorted using fluorescence-activated cell sorting (FACS). RNA and protein analyses will be used to measure the levels of pro-algesic mediators within these two cell types and correlate them with clinical measures of pain. Sensory neurons within the synovial tissue will be imaged to examine their location and proximity to sub-lining fibroblasts. In vitro assays such as neurite outgrowth assays and micro-electrode arrays (MEA) will be used to assess the ability of synovial fibroblasts to sensitise human-induced pluripotent stem cell (hiPSC)-derived sensory neurons.

#### Setting

The study will recruit at two medical centres: Guy’s and St Thomas’ Hospital (GSTT) in London and Nuffield Orthopaedic Centre in Oxford. Patient recruitment will commence in September 2024 and end in September 2027. Patients will be identified by their direct care team through rheumatology outpatient clinics or through review of electronic medical data.

#### Participants

Clinicians or research nurses who are trained in good clinical practice will discuss the study with potential participants at their rheumatology clinic appointment and provide them with a participant information sheet (PIS). Alternatively, if potential participants do not have a clinic appointment in the near future, they can be called by the chief investigator or study delegates, and a PIS can be sent by email or posted to the participants if they show an interest in the study. Participants will be given at least 24 hours to review the PIS before they are contacted for consent. Willing study participants will attend an appointment at a convenient date and time, where written consent will be obtained. Alternatively, consent can be gained virtually, and participants can email the signed consent form to the researcher. Written consent will be obtained after the nature of the study, including potential risks and benefits, is explained to the participants, and the participants have had the opportunity to ask any questions. All participants will retain a copy of their signed consent form, while the original will be kept in the site file.

#### Sample size and rationale

For this study, 50 patients with seropositive RA will be recruited (approximately 25 patients at each site). With n=50, we will have an 80% chance to detect a Spearman’s correlation (LIF vs average knee pain) of 0.36 or larger with alpha=0.05. In the case of under-recruitment, n=40 still has an 80% chance to detect two correlations of r=0.41 or larger at alpha=0.05. In both instances, we will be conducting a one-sided analysis assuming that pain will increase as mediator levels increase.

#### Eligibility criteria (inclusion and exclusion)

##### Subject inclusion criteria

1. Diagnosis of seropositive RA (rheumatoid factor and/or cyclic citrullinated peptide (CCP) positive) meeting 2010 ACR/EULAR classification criteria.
2. Reporting a knee pain numerical rating scale (NRS) ≥3 on a 0–10 scale; we aim to recruit study participants that span a range of knee pain scores.
3. Symptom duration ≥24 months.
4. Stable dose of conventional synthetic disease-modifying antirheumatic drug (DMARD) therapy and/or biologic or targeted synthetic DMARD therapy (preferentially not IL-6 or Janus Kinase (JAK) inhibition) for ≥4 weeks or, if taking steroids, on prednisolone dose ≤10 mg/day or equivalent.

##### Subject exclusion criteria

1. Under 18 years of age.
2. Unable or unwilling to provide informed written consent.
3. Unable to comply with study protocols.
4. Pregnant or breastfeeding.
5. Current or recent (last 90 days) treatment with investigational agents.
6. Knee osteoarthritis ≥grade 2 on the Kellgren and Lawrence classification system on a previous knee X-ray held on the Trust’s medical imaging platform.
7. Diagnosis of fibromyalgia based on ACR 2016 criteria.
8. Treatment with immunosuppressant therapy other than for inflammatory arthritis.
9. Use of intra-articular steroids in the affected knee or intramuscular steroids within the last 4 weeks.
10. Regular use of direct oral anticoagulant therapy, warfarin, high-dose aspirin/clopidogrel ≥150 mg/day or patients with bleeding diathesis.
11. Diagnosis of other painful conditions that could act as a confounding variable, for example, peripheral neuropathy.
12. Use of non-steroidal anti-inflammatories, opioids, gabapentin or pregabalin within 24 hours before assessment.

### Data collection procedures

#### Clinical data collection and pain phenotyping

Demographic and clinical data will be obtained using history and review of electronic records, including age, gender, ethnicity, rheumatoid factor, CCP antibody status, comorbidities, medication, inflammatory markers (within the last 28 days), extra-articular features of RA, smoking status, body mass index and employment status.

A knee pain score using an NRS will be obtained from both knees on three occasions prior to synovial tissue sampling; an average score per knee will be taken forward for analysis. Study participants will be asked the following question for each knee: “*How would you rate your pain on a 0–10 scale in your (left/right) knee, over the last 24 hours, where 0 is* ‘*no pain*’ *and 10 is* ‘*pain as bad as could be*’?” Participants with a knee pain score between 3 (mild pain) and 10 (severe pain) will be recruited to the study.

As a part of their clinical pain phenotyping, participants will complete the short form McGill questionnaire, the PainDETECT questionnaire and the Intermittent and Constant Osteoarthritis Pain (ICOAP) questionnaire. The short form McGill questionnaire is a validated tool used to assess both the quality and intensity of pain.^31^ The PainDETECT questionnaire is a validated questionnaire in RA which will be used to assess for the presence of neuropathic-like pain.^32^ The ICOAP questionnaire will be included to allow comparison to pain data collected from the Restoring Joint Health and Function to Reduce Pain consortium, part of a National Institutes of Health effort to end the opioid public health crisis in the USA.^33^ Additional questionnaires will be used to assess PROMs of anxiety, depression and quality of life, including the Patient Health Questionnaire - Somatic, Anxiety and Depressive Symptoms, the Musculoskeletal Health Questionnaire and the EuroQol 5 Dimensions, 5 Levels (EQ-5D-5L).

As part of our assessment of pain perception, we will use pressure algometry as a measure of central and peripheral sensitisation in our participants. Pain pressure thresholds (ie, the pressure at which the stimulus first becomes painful) will be measured over the medial aspect of the knee (peripheral sensitisation) and at the trapezius (additional central sensitisation) bilaterally. A commercial automated pressure algometer will be applied at a steady rate (approximately 0.5 kPa/s) and the participant will be asked to report ‘*as soon as the usual sensation of pressure changes towards an additional sensation of burning, stinging, drilling or aching’*, at which point, the test will stop and the reading will be recorded.

#### Disease activity scoring

Disease Activity Score of 28 joints with CRP (DAS28CRP) will be used to assess disease activity.

#### Peripheral blood sampling

Blood tests for C-reactive protein (CRP) will be taken unless CRP data from the last 28 days are already available. In addition, up to 50 mL of blood will be taken for research, provided the participant has given consent. Blood will be transferred to the laboratory where peripheral blood mononuclear cells will be isolated (see protocol below).

#### Ultrasound examination of the knee

Musculoskeletal ultrasound will be performed using a LOGIQ E10 (GE HealthCare, Chicago, Illinois, USA) ultrasound machine with a 10–14MHz linear transducer at GSTT and a LOGIQ E9 (GE HealthCare, Chicago, Illinois, USA) ultrasound machine with a GE ML6-15 transducer at Oxford. Ultrasound images will be taken of the knee from the suprapatellar pouch midline, and medial and lateral joint lines. Grey scale (GS) images for synovial thickness and power doppler (PD) images for synovial vascularity will be taken. The PD settings will be adjusted to the lowest permissible pulse repetition frequency to maximise sensitivity and to the maximum colour gain required not to create artefactual noise. The grade of synovial hypertrophy on GS and PD will be recorded, and the knee with the highest grade of inflammation as defined by the standardised Outcome Measures in Rheumatoid Arthritis Clinical Trials (OMERACT) scoring system will be biopsied.^34^

#### X-ray

Knee X-rays taken as part of standard clinical care will be scored using the Kellgren and Lawrence classification system, and patients with a grade ≥2 will be excluded from the study (refer to Exclusion criteria). No additional X-ray will be performed as a part of this research study.

#### Synovial biopsy sampling

The ultrasound-guided biopsies will be performed in a clean procedure room using sterile aseptic technique by suitably trained operators.

Under ultrasound guidance, local anaesthetic will be injected using a 23G needle into the subcutaneous tissue. Using a 19G/21G needle, any fluid will be aspirated and stored in Na-Hep tubes for research purposes with patient consent. Subsequently, 1% lidocaine will be injected inside the joint. A biopsy needle (16G/14G) will be placed within the joint capsule under ultrasound guidance. The maximum tolerated number of biopsies will be taken, with up to 25 biopsies per knee. A sterile dressing will be applied over the biopsy site.

Of all the biopsies, 12 will be placed into a cryo-vial containing 2 mL of CryoStor CS10 (Sigma Aldrich, St. Louis, Missouri, USA) and transferred to the Centre for Inflammation Biology and Cancer Immunology for cryopreservation in liquid nitrogen as described by Donlin *et al*.^35^ Six of the biopsies will be placed in 10% formalin for fixation and paraffin embedding to obtain Krenn scores^36^ for inflammation. Six biopsies will be placed into periodate-lysine-paraformaldehyde (PLP, 75mM lysine monohydrochloride (Sigma-Aldrich, St. Louis, Missouri, USA), disodium hydrogen orthophosphate anhydrous (Sigma-Aldrich, St. Louis, Missouri, USA), 0.2M phosphate buffer (Thermo Scientific, Waltham, Massachusetts, USA), 2% paraformaldehyde (Thermo-Scientific, Waltham, Massachusetts, USA), 5mM sodium-m-periodate (Sigma- Aldrich, St. Louis, Missouri, USA)) buffer and transferred to the Kennedy Institute of Rheumatology, Oxford. One biopsy will be placed on the surface of a T25 culture flask with Dulbecco's Modified Eagle's Limiting Medium (DMEM, Sigma-Aldrich, St. Louis, Missouri, USA) containing 20% fetal bovine serum (FBS, Gibco, Waltham, Massachusetts, USA) which will encourage the migration of fibroblasts from the tissue and thus subsequent expansion and storage of the tissue fibroblasts. If fewer than 25 biopsies are collected, the biopsies will be prioritised for storage in CryoStor CS10.

Participants will be contacted by telephone within 72 hours of the procedure to monitor for adverse events. If an adverse event is suspected, for example, septic arthritis or haemoarthrosis, the patient will be reviewed in person in the rheumatology clinic in a timely manner and appropriate management procedures followed.

### Synovial tissue digestion and cell sorting

The cryopreserved synovial tissue biopsies will be thawed by warming the tube quickly at 37°C in a water bath and transferring the contents immediately into a fresh tube containing digestion buffer (5 mL serum-free Roswell Park Memorial Institute (RPMI, Gibco, Waltham, Massachusetts, USA), 300 µg/mL Liberase TL (Roche, Basel, Switzerland) and 100 µg/mL DNase I (Roche, Basel, Switzerland)). The tube will be placed horizontally into a shaking incubator set at 37°C and 200 rpm for 1 hour and 30 min. After the digestion, the contents of the tube will be passed through a 70 µM strainer and washed with 10 mL RPMI containing 10% FBS, after rinsing the digestion tube first to collect any remaining cells. The cell solution will then be centrifuged at 220×g for 10 min.

After centrifugation, the supernatant will be discarded, and the cell pellet resuspended in 1 mL phosphate-buffered saline (PBS; Gibco,Waltham, Massachusetts, USA) and transferred into a 1.5 mL tube. The tube will be centrifuged at 5900×g at 4°C for 3.5 min and subsequently blocked using human serum albumin for 10 min at room temperature. After blocking, the cells will be washed via centrifugation with PBS and resuspended in the staining mix and incubated at 4°C for 30 min. The staining mix will include antibodies for the following markers: CD90 (marker for sub-lining fibroblasts), podoplanin(PDPN, pan-fibroblast marker), CD45 (immune cell marker), CD146 (mural cell marker), CD31 (endothelial cell marker), CD235a (red blood cell marker), CD3 (T cell marker) and CD14 (myeloid cell marker), in addition to a live/dead dye.

After staining, the cells will be washed via centrifugation with cell sorting buffer (PBS with 0.5% bovine serum albumin (BSA, Sigma-Aldrich, St. Louis, Missouri, USA), 2 mM Ethylenediaminetetraacetic Acid (EDTA, Invitrogen, Carlsbad, California, USA)). Finally, the cells will be resuspended in cell sorting buffer and put on ice ready for FACS.

**Figure 2:**
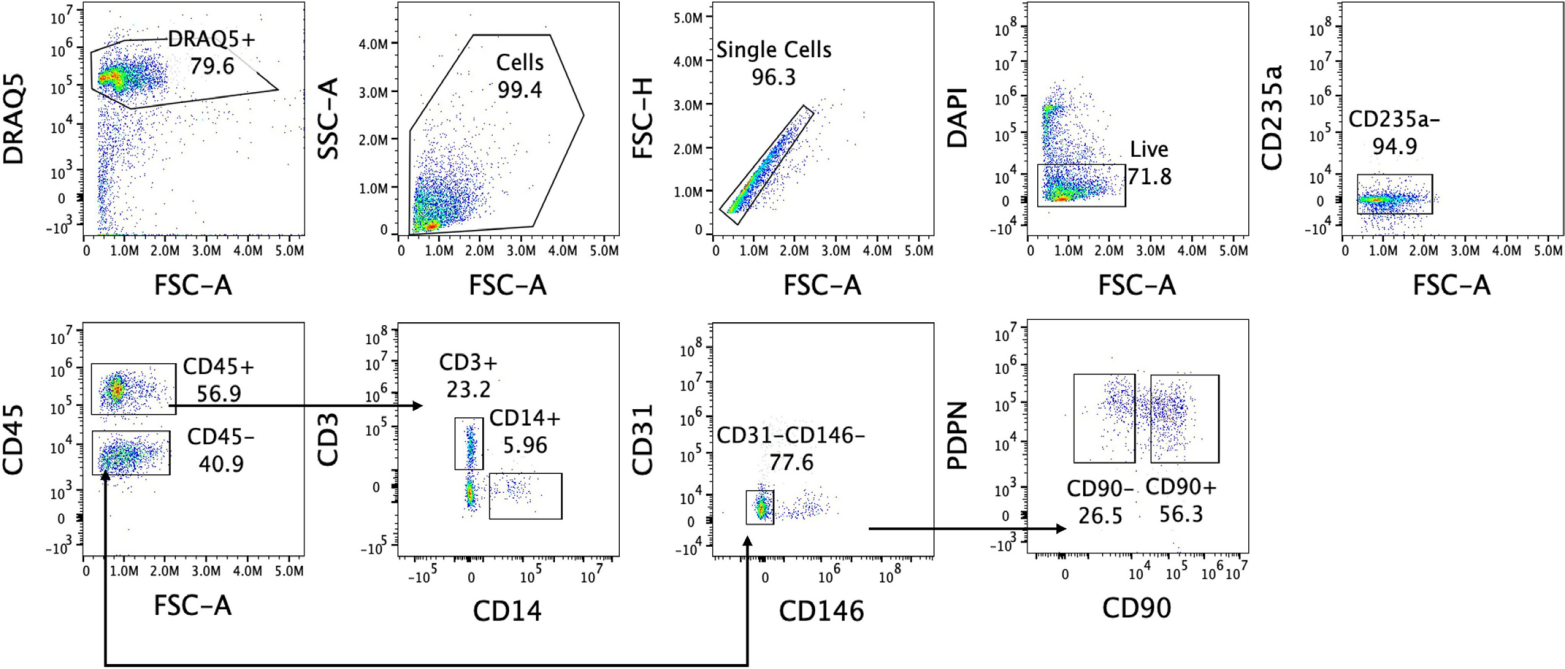
Gating strategy for sorting of podoplanin (PDPN)+CD90+ sub-lining and PDPN+CD90– lining fibroblasts. Deep Red Anthraquinone 5 (DRAQ5) is used to distinguish between cells and debris (first plot), after which the cells are further selected based on their forward scatter (FSC) and side scatter (SSC) (second plot). Single cells are gated (third plot) followed by gating for 4′,6-diamidino-2-phenylindole (DAPI) negative (= live, fourth plot) and CD235a– cells (fifth plot). Immune cells will then be identified using CD45 (sixth plot), followed by gating on CD3+T cells and CD14+ myeloid cells (seventh plot). From the CD45– cells, endothelial and mural cells will be excluded using CD31 and CD146 (eighth plot). The remaining cells are then gated for the two fibroblast (PDPN+) subsets based on their CD90 expression (ninth plot).

To perform FACS, a CytoFlex SRT cell sorter (Beckham Coulter, Brea, California, USA) will be used following the gating strategy depicted in figure 2. PDPN+CD90+ sub-lining and PDPN+CD90− lining fibroblasts will be sorted for both RNA and protein analysis. Additionally, T cells (CD45+CD3+) and myeloid cells (CD45+CD14+) will be sorted for RNA analysis only.

### Formalin fixation and paraffin embedding

Six biopsies will be fixed in formalin and embedded in paraffin. Sections will then be cut and stained with haematoxylin and eosin. Krenn scores will be obtained as described^36^ to assess the degree of tissue inflammation.

### Synovial tissue confocal imaging

Imaging of RA biopsies will be carried out at the Kennedy Institute of Rheumatology, University of Oxford. Six RA synovial biopsies will be fixed in PLP buffer overnight at 4°C, washed three times in sterile PBS before being cryoprotected in 15% sucrose (Scientific Laboratory Supplies, Nottingham, UK) for 24 hours and 30% sucrose for up to 5 days. Following cryoprotection, the slides will be washed three times in PBS before being embedded and frozen in optimal cutting temperature compound (OCT, Tissue-Tek, Sakura Finetek Europe). Sections of 50 µm will be cut onto slides coated in chromium potassium sulphate (Sigma-Aldrich, St. Louis, Missouri, USA) and gelatin (type A, porcine skin, Sigma-Aldrich, St. Louis, Missouri, USA) using a Leica CM3050 S Cryostat (Leica Biosystems, Nussloch, Germany) and left to dry for 24 hours at room temperature. Slides will be rehydrated in PBS 3 times for 5 min. The 50 µm sections will be used for immunofluorescence staining; first, they will be incubated in 1% Sodium Dodecyl Sulfate (SDS, Fisher Bioreagents, USA) solution for 5 min as a brief antigen retrieval step, before being washed three times in PBS. The slides will then be blocked in a buffer consisting of 5% donkey serum (Bio-Rad, USA), human Fc block (Miltenyi Biotec, Germany) and 1X perm/wash buffer (BD Biosciences, SanDiego, California, USA) for 1 hour at room temperature. Slides will then be washed for 5 min in PBS before being stained with 4′,6-diamidino-2-phenylindole (DAPI) for 15 min. The slides will then be washed once again in PBS and incubated with primary antibodies (diluted in 3% BSA in PBS) overnight at 4°C. Primary antibodies include and are not limited to the following: CD31 (mouse, clone WM59, Biolegend, California, USA), protein gene product 9.5 (PGP9.5, rabbit polyclonal, Zytomed, Germany) and calcitonin gene-related peptide (CGRP, sheep, Enzo, New York, USA). They will be applied onto the slides in antibody diluent (3% BSA in PBS). The slides will then be washed in PBS and incubated with secondary antibodies at room temperature for 1 hour. Secondary antibodies include donkey anti-mouse Alexa Fluor 488 (Abcam, Cambridge, UK, ab150153), donkey anti-rabbit 555 (Abcam, Cambridge, UK, ab150074) and donkey anti-sheep 647 (Abcam, Cambridge, UK, ab150179). Slides will then be mounted using ProLong Gold Antifade Mountant (Thermo Fisher, Massachusetts, USA). Images will be obtained using a Zeiss LSM 980 (Germany) confocal microscope.

### Fibroblast explant

One biopsy will be placed on the surface of a T25 flask and allowed a couple of minutes to adhere. Complete DMEM 1 mL containing 20% FBS will slowly be added into the flask in a way that does not detach the tissue from the surface, and the flask will be stored at 37°C. Within a week, the tissue fibroblasts should have begun migrating out of the tissue. At this point, the medium can be changed to standard complete DMEM (10% FBS). Once the fibroblasts become densely packed around the tissue piece, they can be passaged and expanded into a T75 flask. Once the tissue fibroblasts have reached confluency, some will be frozen down in 10% dimethyl sulfoxide(DMSO, Sigma-Alrich, St. Louis, Missouri) plus 90% FBS and the rest will be further expanded for future storage.

### Peripheral blood or synovial fluid mononuclear cell isolation and cryopreservation

Patient-matched blood and synovial fluid (if available) will be taken for isolation and cryopreservation of immune cells, as well as for collection of cell-free serum and synovial fluid.

Peripheral blood mononuclear cells (PBMCs) will be isolated from whole blood using the density gradient centrifugation technique. Initially, blood will be diluted 1:1 with sterile PBS and then carefully layered on top of Lymphoprep (Stem Cell Technologies, Vancouver, British Columbia, Canada) using SepMate (Stem Cell Technologies, Vancouver, British Columbia, Canada) tubes (25 mL per tube). The tubes will be centrifuged at 1200 × g for 10 min, with the brakes off. The PBMCs will then be collected from the interphase and washed via centrifugation with PBS. After washing, the cells will be counted and cryopreserved at a density between 10 million and 20 million cells in 1 mL freezing media (RPMI, FBS and DMSO) in a liquid nitrogen tank.

The serum tube will be left for at least 30 min to ensure the blood has sufficiently clotted. It will then be spun at 2000 × g for 10 min, after which five aliquots will be taken, between 0.2 mL and 1 mL depending on the volume of serum available. The serum aliquots will be stored at −80°C.

Before isolating SFMCs, two 1 mL aliquots of the synovial fluid will be taken and spun at 6000 × g for 3.5 min. The cell-free fluid above the pellet will be aliquoted and stored at −80°C. The remaining synovial fluid will be transferred into a 180 mL sample pot and diluted as required with PBS. The isolation and cryopreservation of cells will then be the same as for PBMC isolation.

### Data analysis

The primary outcome of FiND-Pain RA is to determine whether the average pain score of the biopsied knee correlates with the level of LIF, a putative pain mediator in sub-lining fibroblasts. This will be calculated using a Spearman’s rank correlation, assuming the association is monotone. Bespoke R packages will be used for a non-monotone association.

The secondary outcomes of FiND-Pain RA are to determine whether (1) sub-lining fibroblasts generally produce more pro-algesic mediators compared with lining fibroblasts and whether this is correlated with the average knee pain score of participants; (2) the ratio of sub-lining to lining fibroblasts is higher in individuals who have higher average knee pain scores; (3) there are more sensory nerve fibres in close proximity to sub-lining versus lining fibroblasts; (4) sub-lining fibroblasts (or their mediators) obtained from participants with higher knee pain scores have greater effect on the outgrowth and excitability of hiPSC sensory neurones using neurite outgrowth assays and MEAs; and (5) the levels of pro-algesic mediators correlate with other clinical pain data such as painDETECT scores and pressure algometry.

Once again, we will rely on Spearman’s rank correlations or non-parametric independent sample tests, as appropriate. In instances where we have enough data points to confidently test for and assume a normal distribution, we will use parametric tests.

## Ethics and dissemination

The study has been approved by the Bromley Research Ethics Committee (REC: 21/LO/0712). Results will be disseminated through open-access publications, scientific and clinical conferences, and in partnership with patient involvement groups.

### Trial status

The current protocol is V.4.1 (26 June 2025). Recruitment began in September 2024 and is expected to end approximately in September 2027.
