# Supplementary material for: Fibroblast–Neuron interactions Driving persistent Pain in Rheumatoid Arthritis (FiND-Pain RA) – an observational study protocol
Source: BMJ Open. 2025 Dec 25;15(12):e097892. doi: 10.1136/bmjopen-2024-097892 (PMC12742181; doi:10.1136/bmjopen-2024-097892)
Supplement: Supplementary file 1 [file bmjopen-2024-097892.reviewer_comments.pdf]

PEER REVIEW HISTORY

BMJ Open publishes all reviews undertaken for accepted manuscripts. Reviewers are asked to complete a checklist review form and are provided with free text boxes to elaborate on their assessment. These free text comments are reproduced below.

ARTICLE DETAILS

Title (Provisional)

Fibroblast–Neuron Interactions Driving Persistent Pain in Rheumatoid Arthritis (FiND-Pain RA) – an observational study protocol

Authors

Xenophontos, Mikalena; Baldeweg, Friederike Charlotte; Ross, Rosie; Rutter-Locher, Zoe; Hill, Sarah; Ryan, Sarah; Ali Awadelkareem, Mosab; Law, Shing T; Bennett, David L; Buckley, Christopher D; Humby, Frances; Kirkham, Bruce W; Denk, Franziska; Taams, Leonie

---

VERSION 1 - REVIEW

**Reviewer**	**1**
**Name**	**Rostron, Heather**
**Affiliation**	**Leeds Teaching Hospitals NHS Trust, Children's Research Team**
**Date**	**02-Oct-2025**
**COI**	**None**

A clearly articulated and interesting protocol paper.

Very minor comments:

LIF - please could this be written out in full in the abstract?

Methods section - I wondered if a little more explanation of analysis techniques needed to be described here rather than just describing the outcome measures? Would this be appropriate?

There are no strengths and limitations of the study which would have been nice to see.

Has the study been registered anywhere and has recruitment actually now finished?

Many thanks.

VERSION 1 - AUTHOR RESPONSE

Reviewer Comments: *A clearly articulated and interesting protocol paper. Very minor comments:*

LIF - please could this be written out in full in the abstract?

Apologies for this oversight – this has now been corrected.

Methods section - I wondered if a little more explanation of analysis techniques needed to be described here rather than just describing the outcome measures? Would this be appropriate?

We have added more information on the statistical methods we will use to calculate our primary and secondary outcomes on page 11.

There are no strengths and limitations of the study which would have been nice to see.

These have now been added, in line with BMJ Open Editorial guidelines.

Has the study been registered anywhere and has recruitment actually now finished?

This is not a clinical trial, so the study has not been officially entered into a registry. However, in order to maximise the conclusions that we can draw from our results, we wished to publish this study protocol, as a way to publicly register our primary, hypothesis-testing outcome. Recruitment is still very much ongoing.
